# Utilization of tea polyphenol and cellulose to improve water-soluble porcine liver protein based high internal phase Pickering emulsions stability: interfacial stability and oxidation inhibition

**DOI:** 10.1016/j.fochx.2026.103842

**Published:** 2026-04-07

**Authors:** Yuqian Guan, Yang Wang, Ning Liu, Ying Chen, Cinan Li, Chaoyue Yang, Lihua Shang, Yuanyuan Liu, Qiujin Zhu, Ying Zhou

**Affiliations:** a Guizhou Key Laboratory of New Quality Processing and Storage of Ecological Specialty Food, School of Liquor and Food Engineering, Guizhou University, Guiyang 550025, China; b Key Laboratory Mountain Plateau Animals Genetics and Breeding, Ministry of Education, Guiyang 550025, China

**Keywords:** Water-soluble porcine liver protein, Tea polyphenols, Polyquaternium-10, Interfacial structure, Emulsion stability, Oxidative stability

## Abstract

Water-soluble porcine liver protein (WSLP) shows promise for food applications but is limited by its poor emulsifying stability. This study developed high internal phase Pickering emulsions (HIPPEs) stabilized by a combination of tea polyphenols (TPs), polyquaternium-10 (PQ-10), and WSLP. The incorporation of TPs and PQ-10 collectively enhanced the interfacial wettability of WSLP and promoted structural reorganization, resulting a dense, stable interfacial layer. The resulting emulsions exhibited uniform microstructure, superior viscoelasticity, and prolonged storage stability. Notably, HIPPEs containing 2% PQ-10 achieved the smallest droplet size (33.49 ± 0.95 μm), highest viscosity, and optimal antioxidant activity, with significant reductions in POV and TBARS values (*p* < 0.05). The enhanced stability is attributed to the antioxidant function of TPs and the steric hindrance provided by PQ-10. This work provides insights and a theoretical foundation for designing protein-polyphenol-polysaccharide ternary composites with tailored performance.

## Highlights

• TP and PQ-10 effectively co-stabilize WSLP-based high internal phase Pickering emulsions.
• Enhanced interfacial stability via improved wettability and structural reorganization.
• 4% PQ-10 achieves optimal emulsion performance with superior antioxidant activity.
• Synergistic oxidation inhibition and steric hindrance stabilization mechanism revealed.

## Graphical abstract

**Figure:**
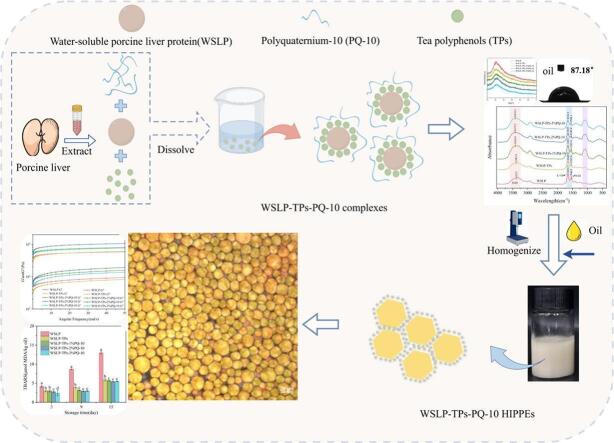

## 1 Introduction

In recent years, food-grade Pickering emulsions have gained increasing interest due to their superior stability, tunable rheology properties, and ability to encapsulate bioactive compounds. These emulsions are stabilized by solid particles adsorbed at the oil-water interface, forming a coalescence-resistant barrier. Although proteins, polysaccharides, and polyphenols each offer distinct advantages, single-component stabilizers often exhibit limited performance. Protein-polysaccharide composites enhance emulsification through complementary mechanisms: proteins provide interfacial activity, while polysaccharides contribute solubility and steric stabilization (Motta, Mulko, Marin, Larrañaga, & Calderón, 2024). Further improvement is achieved in ternary protein-polysaccharide-antioxidant composites, which exhibit superior emulsifying capacity and physicochemical stability. This is especially advantageous in high internal phase Pickering emulsions (HIPPEs, φ > 74%) (Yin et al., 2025), where the self-supporting gel structure of polyhedral droplets require robust interfacial stabilization. Ternary composites not only provide the required mechanical strength and structural cohesion; but also impart antioxidant protection, thereby enhancing the physical and oxidative stability of the emulsion system (Yu et al., 2025). While significant progress has been made in developing Pickering emulsion stabilizers, there remains a need to explore sustainable and cost-effective alternatives derived from agricultural by-products.

According to industry reports, global pork production has exceeded 107.649 million metric tons in previous years (de Souza et al., 2025), thereby generating substantial by-products, including porcine liver. Despite its high nutritional value—rich in iron, zinc, vitamins A and B complex, and high-quality proteins—liver consumption remains largely confined to East and Southeast Asia. Proteins are the second most abundant component in liver after water, and their structural and functional properties are essential for food applications (Narcís Feliu-Alsina & Saguer, 2025). Moreover, these proteins and their enzymatic hydrolysates exhibit physiological benefits, including enhanced iron absorption, immune modulation, and antioxidant activity (López-Pedrouso, Borrajo, Pateiro, Lorenzo, & Franco, 2020). Water-soluble porcine liver protein (WSLP), the most abundant protein in pork liver (76.1%), exhibits excellent foaming, emulsifying, and gelling properties, positioning it as a potential substitute for highly allergenic proteins in food formulations (Feliu-Alsina & Saguer, 2024). Despite these advantages, current emulsifier strategies predominantly rely on plant-based proteins, leaving animal-derived alternatives such as WSLP largely underexplored.

Tea polyphenols (TPs), the principal bioactive constituents in tea, are polyhydroxy phenolic compounds primarily comprising catechins, including epicatechin (EC), epicatechin gallate (ECG), epigallocatechin (EGC), and epigallocatechin gallate (EGCG). These compounds exhibit diverse biological activities, such as antioxidant, anticancer, lipid-lowering, and immunomodulatory effects. EC constitutes the most abundant catechin (60–80% of total TPs), whereas EGCG displays the highest bioactivity (Zhou et al., 2025). The multiple phenolic hydroxyl groups in TPs enable inhibition of lipid oxidation and provide protection against protein oxidation in dispersed systems. Polyphenol nanoparticles can stabilize Pickering emulsions, with TPs-stabilized high internal phase Pickering emulsions maintaining strong antioxidant activity after one-month storage (Tian et al., 2021). However, excessive TPs concentrations may induce protein aggregation and emulsion destabilization (Zhang, Fan, Liu, & Li, 2023). Despite their potent antioxidant capacity, TPs exhibit limited surface activity and poor interfacial barrier formation, requiring combination with stabilizers as antioxidant additives (Tian et al., 2022). Protein-polysaccharide-polyphenol composites demonstrate synergistic enhancement of emulsion stability and bioactivity, highlighting the need for further optimization of TPs application in emulsion systems.

Polysaccharides enhance emulsion stability by providing electrostatic repulsion and steric hindrance at the interface, while increasing the viscosity of aqueous phase to restrict droplet mobility and aggregation. Ternary protein-polysaccharide-polyphenol composites exhibit synergistic enhancement in emulsion stability and bioactivity through the formation of an electrostatically stabilized and sterically hindered interfacial layer, combined with increased continuous phase viscosity (Huang, Luo, Ning, Ye, & Liu, 2024). Numerous studies have demonstrated that Pickering emulsions stabilized by cellulose derivatives retain the excellent stability of surfactant emulsions, while offering additional advantages such as robust stability, good biocompatibility, and low toxicity (Li et al., 2023). Polyquaternium-10 (PQ-10), a cationic cellulose derivative modified with quaternary ammonium groups, exhibits notable antimicrobial activity, hydrophilicity, and mucoadhesive properties (Huang et al., 2023). Our previous study (Wang et al., 2025) revealed that PQ-10, owing to its long-chain structure and positive charge, adsorbs onto protein surfaces to form core-shell structures, thereby suppressing aggregation and promoting uniform droplet formation. This facilitates the development of enhanced elastic networks while improving continuous phase viscosity and steric stabilization. Consequently, we hypothesize that the WSLP-TPs-PQ-10 ternary composite can form a thicker, denser interfacial layer, impart higher viscosity and further enhance emulsion stability.

This study developed high internal phase Pickering emulsions stabilized by tea polyphenols, polyquaternium-10, and WSLP in combination. Comprehensive evaluation of emulsion microstructure, rheological properties, storage stability, and antioxidant performance revealed that the ternary composites formation significantly enhanced interfacial adsorption and stabilized the emulsion system through combined steric hindrance and oxidative protection. These findings not only provide a theoretical foundation for designing animal protein-based ternary composites but also offer practical strategies for developing stable HIPPEs with tailored functionalities, particularly for applications in functional food and nutraceutical delivery systems.

## 2 Materials and methods

### 2.1 Materials

PQ-10 and TPs were purchased from Hubei Xinyu Hong Bio-pharmaceutical Technology Co., Ltd. (Hubei, China), the peanut oil was acquired from Shandong Luhua Group Co., Ltd. (Shandong, China), and the fresh pork livers were purchased from Nantou Huimin Supermarket (Guizhou, China). The temperature was maintained at approximately 4 °C during transportation, and the pork livers were used within one day.

### 2.2 Extraction of WSLP

WSLP was extracted according to previously described methods (Steen et al., 2016; Zouari et al., 2011) with minor modifications. Fresh pork liver was weighed, connective tissues were removed and the liver was minced. The minced liver was homogenized in four volumes of phosphate buffer (0.05 M, pH 7.4) and centrifuged at 10,615×*g* for 20 min at 4 °C using a H2050R centrifuge (rotor radius: 9.49 cm). The supernatant was filtered through gauze to remo*v*e fat, and the precipitate was re-extracted twice. The pooled supernatants were dialyzed (MWCO: 8–14 kDa) and lyophilized to obtain WSLP powder.

### 2.3 Preparation and characterization of WSLP, WSLP-TPs, and WSLP-TPs-PQ-10 composites

The WSLP solution was prepared by dissol*v*ing 2.5 g of WSLP powder in 100 mL of deionized water with stirring until homogeneous, and then stored at 4 °C. Subsequently, 80 mL of this solution was mixed with 0.64 g of TPs (0.8%, w/v) and stirred until complete dissolution to obtain the WSLP-TPs composite solution. Separately, PQ-10 solutions at concentrations of 1%, 2%, and 3% (*w*/*v*) were prepared by hydrating the corresponding mass of PQ-10 powder in deionized water with stirring until homogeneous. The WSLP-TPs composite solution was then mixed with each PQ-10 solution at a volume ratio of 1:1, yielding final PQ-10 concentrations of 1%, 2%, and 3% (w/v), respectively. The resulting composite solutions were designated as WSLP-TPs-1% PQ-10, WSLP-TPs-2% PQ-10, and WSLP-TPs-3% PQ-10, and stored at 4 °C for further analysis.

#### 2.3.1 Detection of Fourier transform infrared (FT-IR) spectroscopy

The molecular structure of the composites was characterized by FT-IR spectroscopy. Lyophilized samples were analyzed with a Nicolet Is20 spectrometer (Thermo Fisher, UK) over a wavenumber range of 400–4000 cm^−1^, according to a previously described method (Wang et al., 2023). The secondary structure composition of WSLP was quantified by peak deconvolution of the amide I region (1700–1600 cm^−1^) using PeakFit (Version 4.12) and SPSS Statistics.

#### 2.3.2 Measurement of X-ray diffraction (XRD) spectrum

The crystalline properties of the samples were measured by an X-ray diffractometer (Rigaku Company, Japan). The sample in Section 2.3 was freeze-dried, ground into a fine powder, and scanned continuously from 10° to 80° (2θ) at a scanning rate of 5°/min.

#### 2.3.3 Measurement of three-phase contact angle

The three-phase contact angles were determined with a DSA25 Contact Angle Goniometer according to the reported method (Tian et al., 2022) with minor modifications. Freeze-dried powders obtained in Section 2.3 were compacted into tablets of approximately 2 mm thickness using a tablet press under a controlled pressure of 15 MPa for 30s, yielding tablets with consistent surface quality. Each tablet was immersed in peanut oil, and a 3 μL droplet of deionized water was deposited onto its surface. Changes in the contact angle were recorded using a high-speed camera equipped with the instrument.

### 2.4 Preparation and characterization of WSLP, WSLP-TPs, and WSLP-TPs-PQ-10 HIPPEs

The high internal phase Pickering emulsions (HIPPEs) were prepared using WSLP-based solutions as the aqueous phase (25% *v*/v) and peanut oil as the oil phase (75% v/v). The aqueous phases consisted of WSLP, WSLP-TPs, and WSLP-TPs-PQ-10 composite solutions, with control groups were prepared by replacing PQ-10 solution with deionized water. Emulsification was performed using an XHF-DY high-speed homogenizer (Ningbo Scientz Biotechnology Co., Ltd., China) at 12,000 r/min for 1 min, with a 20s interval after the first 30s of homogenization.

#### 2.4.1 Optical microscope observation

The morphology of freshly prepared emulsions was examined using a DMEX20 optical microscope (Ningbo Shunyi Instrument Co., Ltd., China). All samples were diluted two-fold with deionized water. A droplet of the diluted emulsion was placed on a microscope slide and coverd with a coverslip. Images were captured at 10× objective magnification.

#### 2.4.2 Determination of droplet size and zeta potential

The droplet size distribution of the emulsions was determined using a LS13320 laser diffraction particle size analyzer (Beckman, USA) according to a modified literature method (Huang et al., 2024). Prior to analysis, emulsions were diluted 40-fold with deionized water to prevent multiple scattering effects.

Zeta potential measurements were measured using a ZEN3600 nano particle size and zeta potential analyzer (Malvern Instruments Ltd., UK). All emulsions were diluted 10 times with deionized water before measurement.

#### 2.4.3 Confocal laser scanning microscopy (CLSM)

The microstructure of the HIPPEs was observed using an LSM900 confocal laser scanning microscope (Zeiss, Germany). The HIPPEs were stained with Nile Red (0.01%) and Nile Blue A (0.1%). Images were acquired at 20× magnification with excitations at 488 nm and 561 nm, respectively, rendering the oil phase green and the aqueous phase red in the obtained micrographs.

#### 2.4.4 Scanning electron microscope (SEM) observation

The microstructure of lyophilized HIPPEs powders prepared from the complexes was characterized using a Sigma 300 scanning electron microscope (ZEISS, Germany) according to a previously described method (Huang et al., 2024) with modifications. To prepare samples for SEM observation, volatile cyclohexane was used as the oil phase instead of peanut oil during emulsion preparation, followed by freeze-drying. An appropriate amount of the freeze-dried sample was mounted on conductive adhesive tape and subsequently sputter-coated with a thin layer of gold. SEM observations were conducted at an accelerating voltage of 3.0 k*V* to examine the morphological features. Porosity analysis was performed on the obtained micrographs using Image J software.

#### 2.4.5 Low-field nuclear magnetic resonance (LF-NMR) and magnetic resonance imaging (MRI)

LF-NMR analysis was performed using an NMI20-025V-1 spectrometer (Niumai Corporation, Suzhou, China) according to a previously reported method (Liu et al., 2025) with minor modifications. Briefly, 5 g of emulsion was placed in a 10 mL glass vial and inserted into a 25 mm diameter glass tube within the instrument. The imaging parameters were set as follows: field of view (FOV) = 100 mm, slice width = 4 mm, slice gap = 0.7 mm, repetition time (TR) = 6000 ms, echo time (TE) = 0.2 ms, and number of averages = 4. The acquired images were processed using NMR image analysis software, with grayscale data were uniformly mapped and converted into pseudo-color images for visualization.

#### 2.4.6 Microrheological behavior of emulsions

The rheological properties of the emulsions were measured at 25 °C using a Mars60 rotational rheometer (Haake, Germany) equipped with 40 mm roughened peltier plates, following a previously described method (Zeng et al., 2025) with slight modifications. The measurement gap was set to 1000 μm, and all samples were allowed to rest for 2 min after loading to ensure stress relaxation and thermal equilibration prior to testing. An amplitude sweep was conducted at a constant angular frequency of 1 Hz over a strain range of 0.01–100% to identify the linear viscoelastic region (LVR). Based on the results, a strain of 1% was confirmed to be within the LVR for all samples and was selected for subsequent frequency sweep tests. Frequency sweep were performed over 0.1–100 rad/s to determine the storage modulus (G′), loss modulus (G″), and loss tangent (tan δ = G″/G′). Apparent viscosity was measured under steady shear conditions at the shear rates from 0.01 to 100 s^−1^.

### 2.5 Emulsion stability analysis

#### 2.5.1 Storage stability

The freshly prepared emulsion was transferred into a glass bottle, sealed it with a lid, and stored it at 4 °C for 35 days, photographs were taken periodically to record the appearance of the emulsion.

#### 2.5.2 Freeze-thaw stability

The freeze-thaw stability of the emulsion was evaluated according to the described method (Wang et al., 2024). Freshly prepared emulsions was frozen at −20 °C for 24 h, thawed at room temperature, and photographed to document their appearance after each cycle.

#### 2.5.3 Centrifugal stability

Freshly prepared emulsion was centrifuged at 2653×*g* for 15 min, and photographed to document their appearance after centrifugation. Centrifugal stability was calculated according to the following equation:

(1) $$\text{Centrifugal stability}\left(\%\right)=\frac{m_{0}-m_{1}}{m_{0}}\times 100\%$$

where *m*_0_ is the weight of the emulsion before centrifugation, and *m*_1_ is the weight of the oil and water separated from the emulsion.

#### 2.5.4 Thermal stability

The thermal stability of the emulsions was evaluated according to the method in the reference (Wang et al., 2025). Freshly prepared emulsions were linearly heated in a water bath to 80 °C, maintained at this temperature for 30 min, cooled to room temperature, stored o*v*ernight, and then centrifuged at 2653×*g* for 15 min. Photographs were taken to record their appearance after centrifugation. Thermal stability was calculated using the following equation:

(2) $$\text{Thermal stability}\left(\%\right)=\frac{m_{2}-m_{3}}{m_{2}}\times 100\%$$

where *m*_2_ is the weight of the emulsion before centrifugation, and *m*_3_ is the weight of the oil and water separated from the emulsion after centrifugation.

### 2.6 Determination of the antioxidant activity of the emulsion

#### 2.6.1 DPPH free radical scavenging rate

Fresh prepared HIPPEs were diluted 40-fold with deionized water. DPPH radical scavenging activity was evaluated according to a previously described method (Guan et al., 2024) with slight modifications. 2 mL of the diluted sample was mixed with 2 mL of DPPH solution (0.2 mmol/L, w/v). The mixture was incubated in darkness for 30 min, and the absorbance was measured at 517 nm. DPPH radical scavenging activity calculated using the following equation:

(3) $$\text{DPPH free scavenging activity}\left(\%\right)=\left(1-\frac{\left(A_{i}-A_{j}\right)}{A_{c}}\right)\times 100\%$$

where *A*_*i*_, *A*_*j*_*,* and *A*_*c*_ represent the absorbance of the test mixture (2 mL sample + 2 mL DPPH solution), the sample background (2 mL sample + 2 mL ethanol), and the reagent blank (2 mL ethanol +2 mL DPPH solution), respectively.

#### 2.6.2 ABTS free radical scavenging rate

ABTS radical scavenging activity was evaluated according to a previously described method (Ke & Li, 2024) with minor modifications. Freshly prepared HIPPEs were diluted 40-fold with deionized water. The working solution was prepared by mixing 7 mmol/L ABTS and 2.45 mmol/L potassium persulfate equally, incubating in the dark for 12 h, and diluting to an absorbance of 0.70 ± 0.02 at 734 nm. For the assay, 1 mL diluted sample was mixed with 3 mL ABTS working solution and incubated in the dark for 60 min, after which the absorbance was measured at 734 nm. ABTS radical scavenging activity was calculated using the following equation:

(4) $$\text{ABTS free scavenging activity}\left(\%\right)=\left(1-\frac{\left(A_{i}-A_{j}\right)}{A_{c}}\right)\times 100\%$$

where *A*_*i*_, *A*_*j*_, and *A*_*c*_ represent the absorbance *v*alues of the test mixture (1 mL sample+3 mL ABTS solution), the sample background (1 mL sample+3 mL ethanol), and the reagent blank (1 mL ethanol+3 mL ABTS solution), respecti*v*ely.

### 2.7 Emulsion oxidation stability

Emulsion samples (10 mL) were stored in sealed 50 mL centrifuge tubes at 50 °C in the dark. TBARS values were measured at predetermined time inter*v*als (3, 9 and 15 days) to monitor the progression of lipid oxidation.

#### 2.7.1 Primary oxidation products

The oxidative stability of the emulsions was evaluated by measuring lipid oxidation products. Peroxide values (POVs), indicative of primary lipid oxidation, were determined according to a previously described method (Wu et al., 2025) with minor modifications. Briefly, 1 mL of emulsion was mixed with 5 mL of isooctane/isopropanol (3:1, v/v) and centrifuged at 10,615×*g* for 10 min at 4 °C. An aliquot of 200 μL of the upper oil phase was reacted with 2.8 mL of methanol/n-butanol (2:1, v/v), followed by the addition of 20 μL each of 3.94 M potassium thiocyanate and ferrous ion solution (prepared by mixing equal volumes of 0.132 M BaCl₂ and 0.144 M FeSO₄). After 20 min of reaction in the dark, the absorbance was measured at 510 nm against a methanol/n-butanol (2:1, v/v) blank. A standard curve (y = 0.0426x + 0.0577, R^2^ = 0.9989) was constructed using hydrogen peroxide. POVs were calculated from the standard curve and expressed as milliequivalents of oxygen per kilogram of oil (meq O₂/kg oil). Samples were measured in triplicate, using an individually prepared sample for each replicate.

#### 2.7.2 Secondary oxidation products

Thiobarbituric acid reactive substances (TBARS), representing secondary oxidation products, were determined according to a previously described method (Zhang, Fan, Liu, & Li, 2022) with minor modifications. Briefly, 1 mL of the emulsion was mixed with 2 mL of thiobarbituric acid (TBA) reagent (15% trichloroacetic acid and 0.375% thiobarbituric acid in 0.25 M HCl). The mixture was heated in boiling water for 15 min, immediately cooled in ice water, and then centrifuged at 10,615×*g* for 10 min. The supernatant was collected, and its absorbance was measured at 532 nm. A standard curve (y = 0.0271x + 0.0776, R^2^ = 0.9997) was constructed using 1,1,3,3-tetraethoxypropane. TBARS values were calculated from the standard curve and expressed as μmol malondialdehyde (MDA) equivalents per kilogram of oil (μmol MDA/kg oil).

### 2.8 Statistical analysis

2.8

All experiments were conducted in triplicate, and the results are expressed as the mean ± standard deviation. Statistical differences were evaluated by one-way analysis of variance (ANOVA) followed by Duncan's multiple range test, with significance accepted at *p* < 0.05. Statistical analysis was performed using the SPSS statistical 25 analysis program, and graphs were plotted using Origin 2024.

## 3 Results and discussion

### 3.1 Structural analysis of the composites

**Fig. 1:**
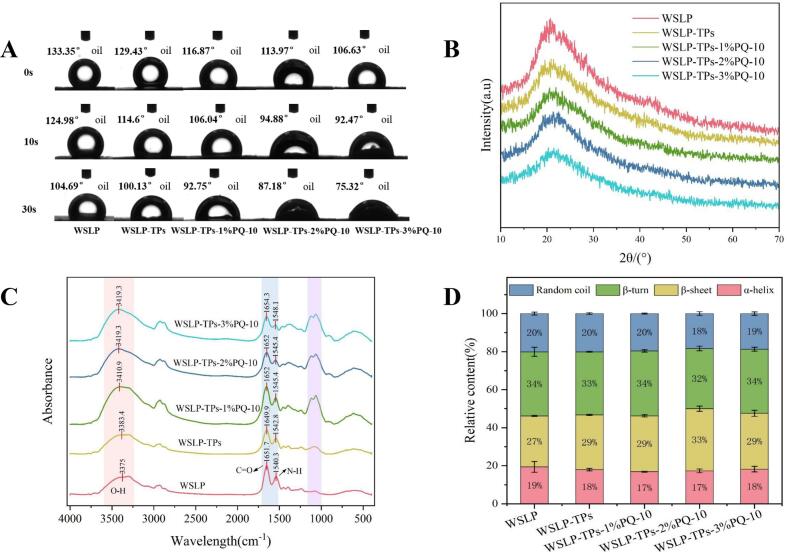
Structural analysis of the composite particles. (A) Three-phase contact angles (θo/w). (B) X-ray diffractograms. (C) Fourier transform infrared (FT-IR) spectra. (D) Secondary structure changes of WSLP within the composites.

The interfacial wettability of composites, quantified by the three-phase contact angle (θ), is a critical determinant of emulsion stability. Optimal amphiphilicity is achieved when θ approaches 90°, facilitating effective interfacial adsorption and formation of a stable interfacial film that enhances separation energy (ΔE) and suppresses droplet coalescence (Wang, Zhang, Yang, Min, & Wang, 2024). As shown in Fig. 1A, The contact angle of WSLP-TPs composite was lower than that of WSLP at all time points, indicating that TPs slightly enhanced surface hydrophilicity through interactions with WSLP. However, this value remained considerably higher than 90°, suggesting suboptimal wettability for O/W stabilization (Sharkawy, Barreiro, & Rodrigues, 2020). As the concentration of PQ-10 increased, the contact angle decreased progressively and significantly. This reduction is attributed to the increased surface density of hydrophilic groups, including quaternary ammonium (–N^+^(CH₃)₃) and hydroxyl moieties, which enhanced water affinity.

XRD analysis was performed to investigate the influence of TPs and varying PQ-10 concentrations on crystalline structure of WSLP. As shown in Fig. 1B, all samples displayed a broad diffraction peak near 21.3°, confirming their amorphous nature (Chen et al., 2024). The incorporation of TPs and PQ-10 markedly reduced peak intensity without shifting peak positions, suggesting preservation of the original crystalline features. This reduction is likely attributed to molecular interactions, indicating hydrogen bonding and electrostatic associations, that disrupt the internal hydrogen-bond network of WSLP, leading to a more disordered protein conformation (İŞÇİMen, E. M., 2025). In summary, these results demonstrate that TPs and PQ-10 promoted amorphization by disrupting the rigid structure of WSLP while leaving its inherent crystalline domains unaffected.

FT-IR analysis was performed to investigate molecular interactions in WSLP-TPs-PQ-10 ternary composites. As shown in Fig. 1C, no new absorption peaks were observed, indicating the absence of chemical reactions and suggesting physical adsorption, primarily through electrostatic interactions, as the dominant complexation mechanism (Liu, Zhao, Li, Meng, & Liu, 2021). All samples displayed a broad amide A band (3600–3000 cm^−1^) corresponding to N-H/O-H stretching vibrations. The hydrogen-bonded peak shifted from 3375 cm^−1^ in WSLP to 3425.6 cm^−1^ in ternary composites, with the extent of blueshift increasing in a concentration-dependent manner with PQ-10 addition. This blueshift indicates a weakening or reorganization of the original hydrogen-bonding network involving N—H groups, likely due to competitive interactions introduced by TPs and PQ-10 (Tao, Zhu, Zhu, Lei, & Zhao, 2024). FT-IR analysis of WSLP reveals characteristic amide I (1652 cm^−1^) and amide II (1548 cm^−1^) bands. The results suggest a redshift of amide II, indicating modified N—H vibrations arising from electrostatic or hydrophobic interactions (Chen et al., 2020). Enhanced absorption in the 1200–1000 cm^−1^ region corresponded to C—O vibrations originating from PQ-10 cellulose and intermolecular hydrogen bonding. Secondary structure analysis (Fig. 1D) showed that the α-helix decreased from 19 ± 2.85% to 17 ± 0.97% (*p* < 0.05), β-turn from 34 ± 0.64% to 32 ± 1.20% (*p* < 0.05), and random coil from 20 ± 0.77% to 18 ± 0.97% (*p* < 0.05). In contrast, the β-sheet content increased markedly from 27 ± 0.23% to 33 ± 1.34% (*p* < 0.05). These structural rearrangements suggest polypeptide chain reorganization and hydrogen bond redistribution within the protein matrix (Liu et al., 2025). These results confirm that WSLP, TPs, and PQ-10 form composites primarily driven by hydrophobic and electrostatic interactions, synergistically assisted by hydrogen bonding.

### 3.2 Analysis of droplet size distribution and zeta potential

**Fig. 2:**
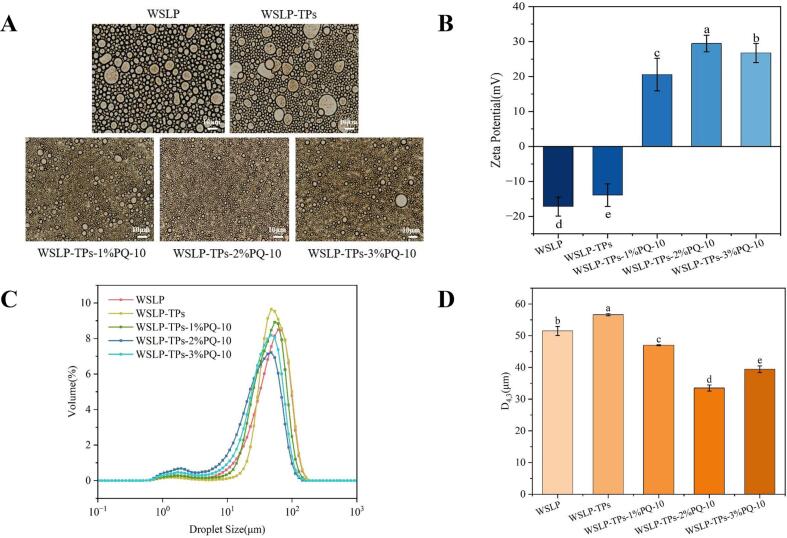
Droplet Morphology, Zeta Potential and Particle Size Distribution of HIPPEs. (A) Optical microscope images of HIPPEs (scale: 10 μm); (B) zeta potential; (C) Droplet size distribution and (D) volume-mean diameter (D[4,3]).

Fig. 2A presents optical micrographs of HIPPEs. All emulsions exhibited spherical droplets, indicating effective interfacial adsorption and film formation (Zhao et al., 2022). WSLP-stabilized emulsions displayed large and polydisperse droplets, likely due to incomplete interfacial coverage. The WSLP-TPs HIPPEs exhibited the largest droplet sizes among all samples, attributed to protein aggregation induced by excess hydroxyl groups that impaired interfacial spreading (Tang, Yang, Wang, & Wang, 2025). Some large droplets displayed non-spherical shapes, possibly resulting from Laplace pressure being exceeded by surrounding droplet compression (Lei et al., 2025). At 2% PQ-10, emulsions achieved the most uniform droplet distribution with smallest mean diameter, confirming that an optimal PQ-10 concentration facilitates droplet fragmentation and interfacial polymer cross-linking, thereby enhancing viscosity and stability (Zheng et al., 2025).

Zeta potential is a key indicator of emulsion stability, with higher absolute values enhancing stability through electrostatic repulsion, while lower values favor droplet aggregation (Niu et al., 2025). As shown in Fig. 2B, the zeta potential of WSLP HIPPEs was negative, indicating a negatively charged surface. Upon the addition of TPs alone, the zeta potential remained negative but shifted toward less negative values, indicating reduced surface charge due to conformational rearrangement and charge shielding from non-covalent interactions or covalent cross-linking between TPs and WSLP (Miao et al., 2026). The addition of PQ-10 markedly increased the absolute zeta potential, reaching a maximum at 2% PQ-10, reflecting enhanced interfacial adsorption density and the formation of a compact interfacial membrane. This reinforcement is attributed to electrostatic adsorption of cationic PQ-10 onto the anionic WSLP-TPs composite, forming a dense three-dimensional network at the interface that acts as a physical barrier against coalescence through combined steric hindrance and electrostatic repulsion (Cui et al., 2024).

According to Stokes' law, emulsion stability is governed by droplet size and continuous phase viscosity, with smaller droplets generally enhancing stability. Fig. 2C and D illustrate the droplet size distribution of composite emulsions. WSLP-stabilized HIPPEs exhibited the largest average droplet size, likely due to the poor amphiphilic wettability of WSLP. In contrast, the WSLP-TPs HIPPEs showed a narrower but right-shifted distribution, indicating an increased proportion of larger droplets. Incorporation of PQ-10 shifted the distribution leftward and reduced the volume-weighted mean diameter, reflecting the formation of finer droplets. At 2% PQ-10, the population of small droplets peaked and the mean diameter reached its minimum, attributed to the construction of a dense interfacial network by composite nanoparticles that enhances emulsion stability (Ravera, Dziza, Santini, Cristofolini, & Liggieri, 2021). A further increase in PQ-10 concentration to 3% led to a subsequent increase in droplet size, consistent with the observations from microscopy.

### 3.3 Microscopic structural analysis of emulsions

**Fig. 3:**
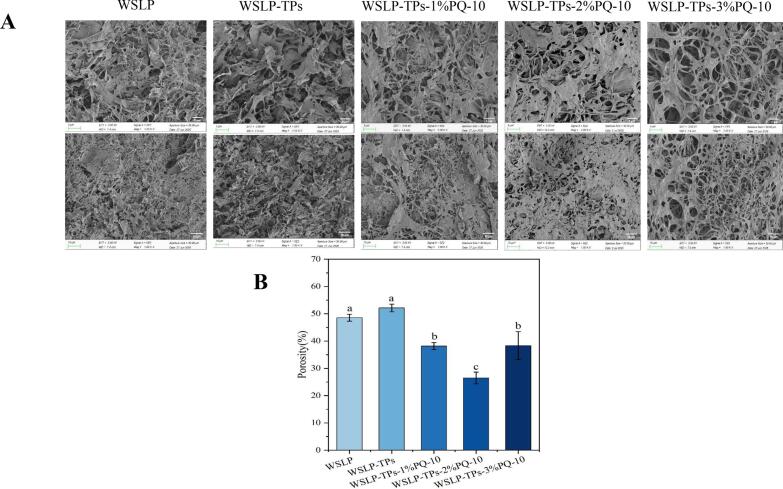
Microstructure of HIPPEs. (A) SEM images of the freeze-dried HIPPEs, showing a low-magnification (top, 1000×) and a high-magnification (bottom, 2000×) view and (B) quantitative analysis of the porosity derived from the SEM images. Different lowercase letters above the bars indicate significant differences (*P* < 0.05).

SEM imaging of the HIPPEs (Fig. 3A) revealed a porous microstructure resulting from the removal of water and cyclohexane during freeze-drying. Porosity, quantified from SEM image, is shown in Fig. 3B. WSLP-stabilized HIPPEs exhibited a collapsed network structure with loose, heterogeneous, and high porosity. Upon the addition of TPs alone, porosity and structural looseness further increased, likely due to insufficient emulsifier coverage at the oil-water interface, leading to a weakened interfacial film and enhanced droplet coalescence (Kim, Zeng, Velikov, & Velev, 2026). Addition of PQ-10 resulted in a rough, fibrous network with uniform pores and significantly reduced porosity, decreasing from 52.14% ± 1.40% to 24.69% ± 2.17% (*P* < 0.05). This structural refinement is attributed to the formation of a denser and more rigid interfacial film by PQ-10, providing steric hindrance that suppressed droplet aggregation and enhanced emulsion stability (Feng et al., 2020). The emulsion containing 2% PQ-10 exhibited the lowest porosity, consistent with the formation of a compact and uniform three-dimensional network. In contrast, increasing the PQ-10 concentration to 3% led to increased porosity and larger droplet sizes, in agreement with optical microscopy and particle size analysis.

**Fig. 4:**
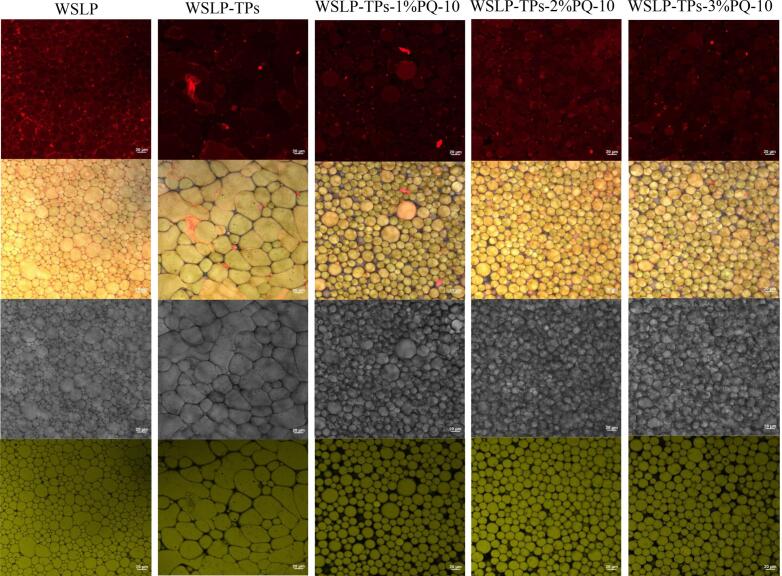
Confocal laser scanning microscopy (CLSM) images of HIPPEs. Aqueous phase stained with Nile Blue A (shown in red), oil phase stained with Nile Red (shown in green) (scale: 20 μm). (For interpretation of the references to color in this figure legend, the reader is referred to the web version of this article.)

CLSM is employed to visualize the morphological characteristics of emulsions. As shown in Fig. 4, the green oil droplets were surrounded by the red aqueous phase, confirming that all prepared emulsions were of the oil-in-water (O/W) type. The droplets exhibited polyhedral structures resulting from mutual compression under high internal phase conditions and displayed a closely packed arrangement. The red fluorescence surrounding the oil droplets confirmed interfacial adsorption of WSLP, the WSLP-TPs binary composite, and the WSLP-TPs-PQ-10 ternary composite at the oil-water interface, forming a protective layer (Cai, Zhang, & Xie, 2024). With increasing PQ-10 concentration, droplet size progressively decreased and size distribution became more uniform, consistent with optical microscopy observations (Fig. 2A) and particle size measurements (Fig. 2C). The formation of a thickened, uniform interfacial layer likely enhanced the interfacial barrier properties, providing an effective spatial barrier against droplet aggregation and thereby improving emulsion stability (Zhao, Liang, Wang, Zu, & Wang, 2024). In contrast, WSLP-stabilized HIPPEs showed larger, heterogeneous droplets, while those of WSLP-TPs HIPPEs exhibited irregular morphologies. This observation suggests inadequate interfacial elasticity and structural instability, likely arising from protein–polyphenol interactions that disrupt droplet integrity during homogenization (Shu et al., 2025). With increasing PQ-10 concentration, the droplets became closely packed into regular polyhedral structures, reflecting enhanced emulsifying capacity and interfacial film formation. This enhancement is attributed to the long-chain cationic structure of PQ-10, which facilitates electrostatic adsorption onto negatively charged protein-polyphenol composites. The resulting intermolecular cross-linking improves interfacial coverage and reinforces the mechanical strength of the interfacial layer, thereby suppressing droplet coalescence while enhancing continuous phase viscosity and steric stabilization (Luo et al., 2025). At 2% PQ-10, the oil droplets exhibited the smallest size, homogeneous distribution, and close packing, indicating the formation of a dense interfacial layer with high stability. In contrast, at 3% PQ-10, the localized appearance of larger droplets suggested interfacial supersaturation due to excess stabilizer.

### 3.4 Analysis of LF-NMR and MRI

**Fig. 5:**
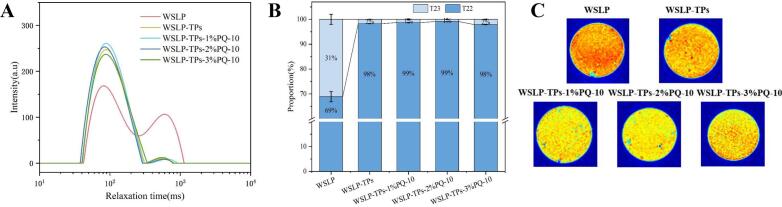
Water distribution of HIPPEs. (A) T_2_ relaxation time curves and (B) their area proportion of various peaks; (C) magnetic resonance imaging images. Different lowercase letters above the bars indicates significant differences. (P < 0.05) (Wang et al., 2024).

LF-NMR analysis was employed to investigate the water distribution within the emulsions. This technology characterizes the chemical environment of water molecules by detecting the relaxation properties of protons (^1^H) in water. Fig. 5A and B show the relaxation times and corresponding peak area proportions of immobilized water (T₂₂) and free water (T₂₃) components. After the addition of TPs alone, the proportion of the T_23_ component significantly decreased from 31 ± 2.05% to 1.77 ± 0.03% (*p* < 0.05), attributed to the binding of free water by hydrophilic phenolic hydroxyl groups in TPs, which promoted its conversion to immobilized water. With increasing PQ-10 concentration, the T_23_ relaxation time in the emulsion shifted toward shorter values, indicating that the synergistic interaction between TPs and PQ-10 promoted the formation of a denser gel microstructure. This structural enhancement improved water immobilization within the network and contributed to improved emulsion stability (Wang et al., 2024).

Pseudo-color MRI images (Fig. 5C) visually represent the hydration state of the emulsions. Signal intensity correlates with regional water content: brighter red areas represent higher moisture, whereas darker blue regions correspond to lower hydration. Compared with the WSLP group, the introduction of TPs resulted in a noticeable reduction in bright red domains, suggesting that the hydrophilic phenolic hydroxyl groups of TPs interacted with free water molecules and promoted their conversion to an immobilized state, thereby enhancing water entrapment within the protein matrix. The WSLP-TPs-2%PQ-10 HIPPEs exhibited uniform water distribution and superior water-holding capacity, demonstrating enhanced moisture regulation and structural stability. This enhancement is attributed to the effective adsorption of PQ-10 onto the surfaces of the WSLP-TPs HIPPEs, which reinforced steric hindrance and electrostatic repulsion between droplets, thereby inhibiting oil droplet re-aggregation (Tang, Gao, Zhang, & Tang, 2021). These findings were corroborated by SEM and CLSM analyses. SEM images (Fig. 3A) revealed a highly porous network with increased void space, indicating enhanced water encapsulation capacity, while CLSM images (Fig. 4) exhibited markedly diminished red aqueous regions, confirming water immobilization. Collectively, these structural changes contributed to a significant reduction in free water content, further supporting the improved stability of the composite emulsion at the microscopic level.

### 3.5 Analysis of the rheological properties of the emulsion

**Fig. 6:**
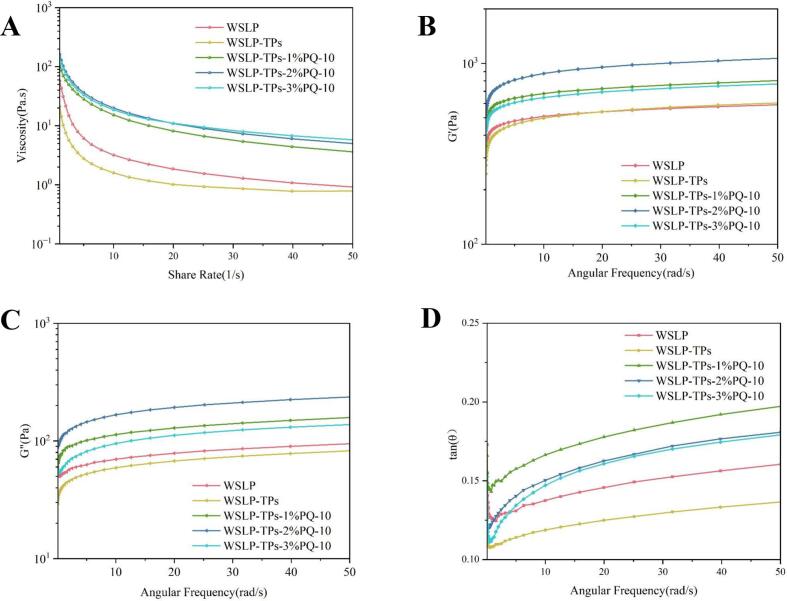
Rheological properties of HIPPEs. (A) Apparent viscosity. (B) Loss factor (tan δ = G″/G′). (C) Storage modulus (G′) and (D) loss modulus (G″). Different lowercase letters indicate significant differences (P < 0.05).

The rheological properties of the emulsions, which are critical determinants of stability and functionality, are presented in Fig. 6A–D. All emulsions displayed shear-thinning behavior, with viscosity decreasing over the shear rate range of 0.1–100 s^−1^ (Fig. 6A). Incorporation of PQ-10 significantly increased apparent viscosity and attenuated shear-thinning, indicating enhanced steric hindrance that suppresses droplet coalescence and improves physical stability. This behavior is attributed to two mechanisms (Mao et al., 2025): (i) electrostatic interaction between PQ-10 and proteins that form a three-dimensional network providing steric stabilization; and (ii) reduced droplet spacing that strengthens van der Waals forces, promoting weak gelation. At 3% PQ-10, a decrease in apparent viscosity was observed, coinciding with the concentration regime where excess unadsorbed polymer chains dominate the continuous phase, leading to weakened inter-droplet interactions and reduced flow resistance.

As shown in Fig. 6B and C, the storage modulus (G′) and loss modulus (G″) of the emulsions were measured, representing the solid-like and liquid-like viscoelastic characteristics, respectively. Across the frequency range of 0.1–100 rad/s, G′ consistently exceeded G″, indicating the formation of a strong network structure with predominantly elastic behavior (Lai et al., 2022). Both G′ and G″ increased with angular frequency. Notably, the emulsion containing 2% PQ-10 exhibited the highest G′ and G″ values, reflecting the optimal network density and structural integrity. In contrast, when the PQ-10 concentration was increased to 3%, both moduli decreased. This reduction is attributed to depletion flocculation induced by unadsorbed PQ-10 chains in the continuous phase, which generate osmotic pressure that promotes droplet aggregation and disrupts the homogeneous three-dimensional network, thereby compromising the viscoelastic properties and overall stability of the emulsion (Tardy et al., 2021).

The loss factor (tan δ = G″/G′) was used to further characterize the viscoelastic behavior. An increase in tan δ indicates enhanced viscous response, whereas a decrease reflects more dominant elasticity (Deng et al., 2025). As shown in Fig. 6D, the tanδ values remained consistently below 1 across all samples, confirming the predominant elastic nature of the emulsions. For all formulations, tan δ increased, suggesting greater energy dissipation under higher deformation rates. The WSLP-TPs-PQ-10 HIPPEs exhibited higher viscosity and more pronounced solid-like behavior, likely associated with the formation of a stronger three-dimensional network in the continuous phase that contributes to enhanced interfacial stability.

### 3.6 Analysis of the physical stability of emulsion

**Fig. 7:**
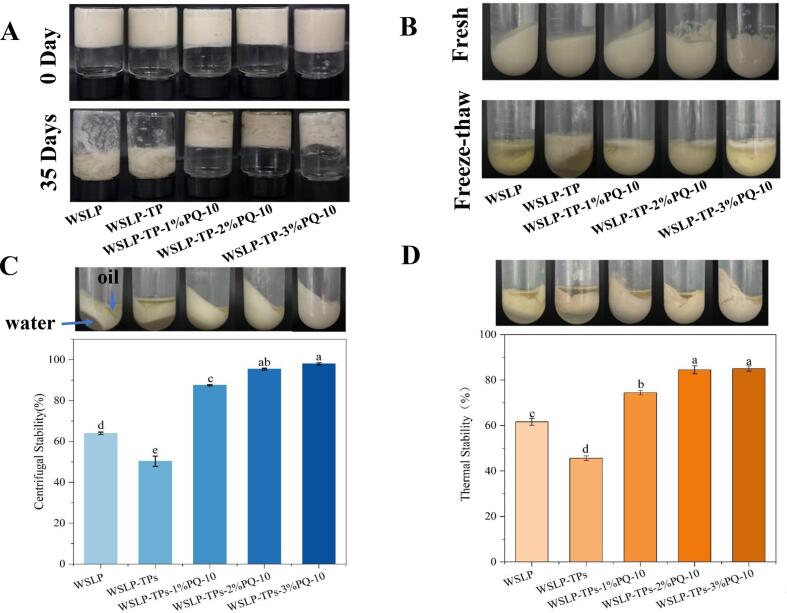
Appearance of different HIPPEs after storage (A) and freeze-thaw (B).HIPPEs stability after centrifugation (C).HIPPEs stability after heating (D). Different lowercase letters indicate significant differences (P < 0.05).

After 35 days of storage at 4 °C, all emulsions showed phase separation (Fig. 7A). WSLP and WSLP-TPs HIPPEs showed severe oil and water release and became fluid upon inversion. Increasing PQ-10 concentration markedly reduced exudation and maintained solid-like states, attributable to a finer droplet size distribution and a stable three-dimensional network that suppressed sedimentation and Brownian motion, coupled with increased viscosity. Notably, at 3% PQ-10, slightly greater oil exudation was observed compared to the 2% formulation, suggesting an optimal concentration threshold for long-term stability. This trend reflects the balance between sufficient network formation and excessive polymer-induced depletion effects (Pan et al., 2020).

Fig. 7B presents the appearance of the HIPPEs after freeze-thaw treatment. All emulsions exhibited varying degrees of phase separation. WSLP-stabilized HIPPEs underwent substantial structural collapse, attributed to ice crystal growth that induced protein aggregation and denaturation, thereby deforming the oil-water interface (Tu, Zhang, & Wang, 2023). Freeze-denaturation of WSLP may also disrupt intermolecular interactions and alter protein conformation, compromising interfacial stability (Zhang et al., 2023). Upon the addition of TPs alone, emulsion instability was further exacerbated, likely due to the inherently compromised interfacial integrity of the binary system. In contrast, incorporation of PQ-10 substantially reduced water and oil release, indicating enhanced freeze-thaw stability. This improvement is attributed to the ability of PQ-10 to reinforce the network structure and mitigate ice crystal disruption, consistent with its stabilizing role observed under other stress conditions (Wang, Zhang, Wang, Xu, & Jiang, 2020).

Centrifugal stability was evaluated by measuring the mass of the remaining emulsion after centrifugation (Fig. 7C). All emulsions underwent phase separation, with the extent varying significantly by composition. WSLP-TPs HIPPEs released substantial oil and water, whereas increasing PQ-10 concentration progressively reduced water separation, improving centrifugal stability from 63.99 ± 0.61% to 98.01 ± 0.54% (*p* < 0.05). At 3% PQ-10, no water separation was observed, indicating exceptional water retention. This enhancement is attributed to three synergistic mechanisms of PQ-10: (i) its emulsifying capacity promotes interfacial particle adsorption, forming a robust steric barrier against droplet coalescence (Xu et al., 2023); (ii) it modulates the rheological properties of the interfacial layer, increasing resistance to external stress; and (iii) capillary effects within the interfacial network of the continuous phase contribute to water retention (Zhang et al., 2025), consistent with LF-NMR data showing increased immobilized water content at elevated PQ-10 concentrations.

The thermal stability of the Pickering emulsions is shown in Fig. 7D. All samples exhibited phase separation after heating and centrifugation. Compared to the WSLP-stabilized system, addition of TPs alone increased oil and water release, reducing thermal stability from 61.62 ± 0.89% to 45.45 ± 0.58% (*p* < 0.05), attributed to TPs-induced protein aggregation that compromised interfacial integrity. In contrast, incorporation of PQ-10 reduced oil separation and led to a concentration-dependent decrease in water release, with thermal stability progressively increasing to 74.45 ± 0.98% (1% PQ-10), 84.54 ± 1.76% (2% PQ-10), and 85.07 ± 1.13% (3% PQ-10) (*p* < 0.05). No significant difference in oil separation was observed between emulsions containing 2% and 3% PQ-10, indicating a plateau at higher concentrations consistent with centrifugal stability results. This enhancement is attributed to two complementary mechanisms: (i) heat-induced denaturation and aggregation of WSLP within the PQ-10 network, which reinforces gel structure and improves phase entrapment (Dai et al., 2025); and (ii) the robust three-dimensional network formed by PQ-10 in the continuous phase, providing physical resistance against thermally induced droplet coalescence and phase migration. The concentration-dependent trend plateauing at 2–3% PQ-10 aligns with the optimal interfacial coverage and network density identified in previous sections.

### 3.7 Analysis of the antioxidant activity of the emulsion

**Fig. 8:**
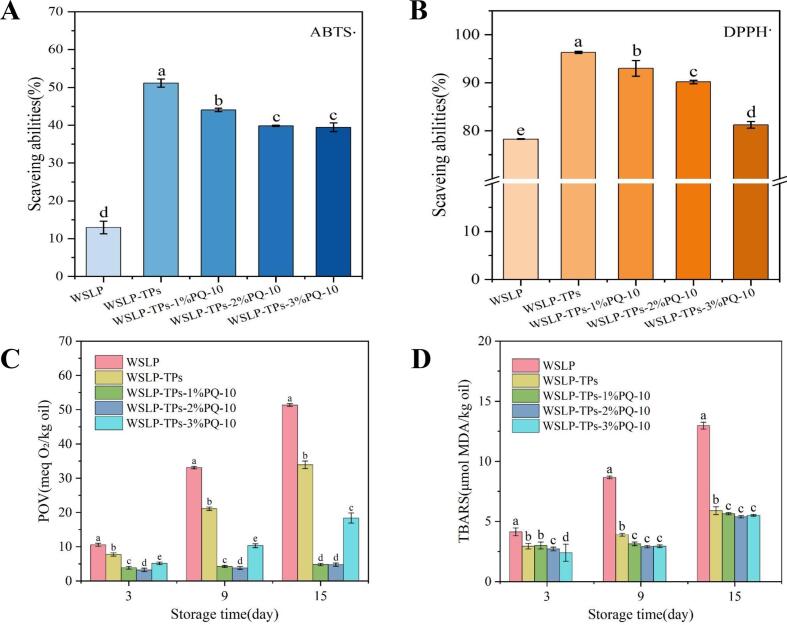
Oxidative stability of HIPPEs. (A) DPPH free radical scavenging rate. (B) ABTS free radical scavenging rate. (C, D) POVs and TBARS values during 15 days of storage at 55 °C. Values are mean ± SD (*n* = 3).

The antioxidant capacity of HIPPEs was evaluated using DPPH and ABTS assays (Fig. 8A and B). The WSLP-TPs HIPPEs exhibited significantly enhanced radical scavenging activity compared to WSLP-stabilized emulsions, attributed to the hydrogen-donating phenolic hydroxyl groups of TPs, which stabilize free radicals (Li et al., 2025). Incorporation of PQ-10 led to a slight decrease in scavenging efficiency; however, the emulsions retained high antioxidant activity. This phenomenon may be explained by the increased viscosity of the continuous phase upon PQ-10 addition, which enhances steric hindrance and slows the diffusion of free radicals within the system. Meanwhile, the formation of a viscoelastic protective barrier at the interface increases the thickness and mechanical strength of the interfacial layer, thereby contributing to improved antioxidant protection (Di, Li, Qin, Wang, & Liu, 2024). At low PQ-10 concentrations, minimal restriction on molecular mobility allowed high scavenging efficiency, whereas higher concentrations promoted emulsion stability while maintaining stabilized antioxidant performance (Yang et al., 2023).

### 3.8 Analysis of the oxidative stability of emulsion

The oxidative stability of HIPPEs was evaluated under accelerated oxidation at 50 °C by monitoring peroxide values (POVs) and thiobarbituric acid reactive substances (TBARS). As shown in Fig. 8C, POVs increased over time in all emulsions, reflecting progressive lipid oxidation. WSLP-stabilized HIPPEs exhibited significantly higher POVs than other formulations, highlighting the critical role of TPs in suppressing interfacial lipid oxidation (Liang et al., 2024). The phenolic hydroxyl groups of TPs donate hydrogen atoms to lipid free radicals and facilitate the scavenging of reactive species at the oil–water interface, thereby delaying peroxidation (Zhang, Chen, Li, Mi, & Li, 2025). The WSLP-TPs-2%PQ-10 HIPPEs demonstrated the strongest antioxidant capacity. This enhanced antioxidant capacity may be attributed to non-covalent interactions among protein molecules, TPs, and PQ-10, which promote interfacial adsorption of WSLP and TPs.

TBARS were commonly used to quantify secondary lipid oxidation products in foods, particularly aldehydes such as malondialdehyde (MDA), a major end product of lipid oxidation. As shown in Fig. 8D, the evolution of secondary oxidation products during accelerated oxidation revealed that WSLP-stabilized HIPPEs exhibited the highest TBARS values, followed by WSLP-TPs HIPPEs. TBARS values progressively decreased with increasing PQ-10 concentration, with the WSLP-TPs-2%PQ-10 HIPPEs showing the lowest value, indicating superior antioxidant stability throughout storage. Collectively, these results demonstrate that the combination of TPs and 2% PQ-10 effectively inhibited both the formation of primary oxidation products (peroxides) and the generation of secondary oxidation products. The improved oxidative stability can be attributed to multiple factors (Atarian, Rajaei, Tabatabaei, Mohsenifar, & Bodaghi, 2019): (i) incorporation of cellulose enhanced emulsion viscosity, thereby impeding the migration of free radicals and metal ions; and (ii) WSLP, TPs, and PQ-10 collectively formed a three-dimensional network through intermolecular interactions, which served as a physical barrier, effectively restricting oxygen permeation and lipid diffusion, thus maximizing oxidative suppression.

## 4 Conclusion

This study developed high internal phase Pickering emulsions (HIPPEs) stabilized by tea polyphenols (TPs), polyquaternium-10 (PQ-10), and WSLP. Interfacial wettability and structural analyses confirmed that TPs and PQ-10 enhanced emulsion stability through hydrophobic interactions, electrostatic adsorption, and hydrogen bonding. Systematic characterization of microstructure, rheological properties, and storage stability revealed that an optimal PQ-10 concentration (2%) significantly improved emulsification performance, yielding smaller droplet size, higher viscosity, and superior interfacial properties. Lipid oxidation analysis of the emulsion demonstrated that the combination of TPs and 2% PQ-10 markedly improved oxidative stability, reducing POV from 51.37 ± 0.40 to 4.78 ± 0.46 (*p* < 0.05) meq O₂/kg oil and TBARS from 12.96 ± 0.28 to 5.40 ± 0.09 (*p* < 0.05) μmol MDA/kg. The enhanced physical and oxidative stability is attributed to the antioxidative function of TPs and the steric stabilization provided by PQ-10. This work provides insights and a theoretical foundation for designing protein–polyphenol–polysaccharide ternary composites with tailored performance.

## CRediT authorship contribution statement

**Yuqian Guan:** Writing – original draft, Visualization, Formal analysis, Conceptualization. **Yang Wang:** Writing – review & editing, Visualization, Supervision, Formal analysis. **Ning Liu:** Writing – review & editing, Methodology, Formal analysis, Conceptualization. **Ying Chen:** Writing – review & editing, Data curation. **Cinan Li:** Writing – review & editing, Investigation. **Chaoyue Yang:** Writing – review & editing, Conceptualization. **Lihua Shang:** Software, Formal analysis, Data curation. **Yuanyuan Liu:** Validation, Methodology. **Qiujin Zhu:** Visualization, Supervision, Methodology, Formal analysis. **Ying Zhou:** Writing – review & editing, Visualization, Supervision, Resources, Funding acquisition, Formal analysis, Conceptualization.

## Declaration of competing interest

The authors declare that they have no known competing financial interests or personal relationships that could have appeared to influence the work reported in this paper.

## Data Availability

Data will be made available on request.
